# Aqua­bis[*N*′-(2-hydroxy­benzyl­idene)isonicotinohydrazide-κ*N*]silver(I) nitrate

**DOI:** 10.1107/S1600536810005027

**Published:** 2010-02-13

**Authors:** Shahriar Ghammamy, Hajar Sahebalzamani, Nina Khaligh, Rahmatollah Rahimi

**Affiliations:** a Department of Chemistry, Faculty of Science, Imom Khomeini International University, Ghazvin, Iran; b Department of Chemistry, Faculty of Science, Islamic Azad University Ardebil Branch, Ardebil, Iran; c Faculty of Chemistry, Iran University of Science and Technology, Tehran, Iran

## Abstract

In the title compound, [Ag(C_13_H_11_N_3_O_2_)_2_(H_2_O)]NO_3_, two N atoms from two pyridine rings of two *N*′-(2-hydroxy­benzyl­idene)isonicotinohydrazide ligands coordinate to the Ag^I^ atom, forming a nearly linear geometry with an N—Ag—N angle of 171.63 (6)°; a water O atom is located at the apical site, completing the T-shaped coordination. The crystal structure is stabilized by extensive O—H⋯O, O—H⋯N and N—H⋯O hydrogen bonding.

## Related literature

For factors affecting the coordination geometry of silver, see: Dong *et al.* (2004 ▶); Niu *et al.* (2009*a* ▶); Sumby & Hardie (2005 ▶); Abu-Youssef *et al.* (2007 ▶). For related structures, see: Li *et al.* (2006 ▶); Näther & Beck (2004 ▶); Niu *et al.* (2009*b* ▶).
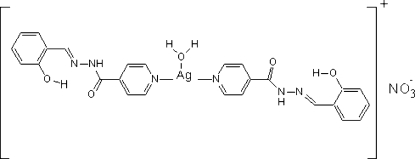


## Experimental

### 

#### Crystal data

[Ag(C_13_H_11_N_3_O_2_)_2_(H_2_O)]NO_3_
*M* *_r_* = 670.39
Monoclinic, 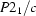

*a* = 11.7194 (6) Å
*b* = 12.6459 (6) Å
*c* = 18.5719 (9) Å
β = 104.738 (1)°
*V* = 2661.8 (2) Å^3^
*Z* = 4
Mo *K*α radiation
μ = 0.82 mm^−1^
*T* = 120 K
0.55 × 0.45 × 0.30 mm

#### Data collection

Bruker SMART 1000 CCD area-detector diffractometer
Absorption correction: multi-scan (*SADABS*; Sheldrick, 1998 ▶) *T*_min_ = 0.686, *T*_max_ = 0.791
26832 measured reflections
6427 independent reflections
5518 reflections with *I* > 2σ(*I*)
*R*_int_ = 0.026

#### Refinement

*R*[*F* ^2^ > 2σ(*F* ^2^)] = 0.029
*wR*(*F* ^2^) = 0.077
*S* = 1.07
6427 reflections
403 parameters
H atoms treated by a mixture of independent and constrained refinement
Δρ_max_ = 1.04 e Å^−3^
Δρ_min_ = −0.57 e Å^−3^

### 

Data collection: *SMART* (Bruker, 2007 ▶); cell refinement: *SAINT-Plus* (Bruker, 2007 ▶); data reduction: *SAINT-Plus*; program(s) used to solve structure: *SHELXS97* (Sheldrick, 2008 ▶); program(s) used to refine structure: *SHELXL97* (Sheldrick, 2008 ▶); molecular graphics: *SHELXTL* (Sheldrick, 2008 ▶); software used to prepare material for publication: *SHELXTL*.

## Supplementary Material

Crystal structure: contains datablocks I, global. DOI: 10.1107/S1600536810005027/pv2256sup1.cif

Structure factors: contains datablocks I. DOI: 10.1107/S1600536810005027/pv2256Isup2.hkl

Additional supplementary materials:  crystallographic information; 3D view; checkCIF report

## Figures and Tables

**Table 1:** Hydrogen-bond geometry (Å, °)

*D*—H⋯*A*	*D*—H	H⋯*A*	*D*⋯*A*	*D*—H⋯*A*
O1*W*—H1*W*⋯O7^i^	0.84 (3)	2.01 (3)	2.844 (2)	171 (3)
O1*W*—H2*W*⋯O2^ii^	0.79 (3)	2.04 (3)	2.821 (2)	172 (3)
N2—H2*N*⋯O6	0.84 (3)	2.09 (3)	2.880 (2)	157 (2)
N5—H5*N*⋯O7^iii^	0.90 (3)	1.97 (3)	2.863 (2)	169 (2)
O2—H2*O*⋯N3	0.85 (3)	1.79 (3)	2.560 (2)	150 (2)
O4—H4*O*⋯N6	0.81 (2)	1.86 (2)	2.607 (2)	153 (2)

Symmetry codes: (i) 
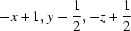
; (ii) 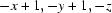; (iii) 
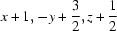
.

**Fig. 1.**
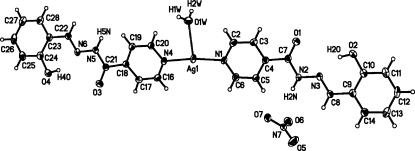
The asymmetric unit of (I) showing the atom-labelling scheme. Displacement ellipsoids are drawn at the 30% probability level.

## supplementary crystallographic information

### Comment

It is noteworthy that the coordination geometry of the silver metal center can be affected by many factors, such as coordination nature of organic ligands, temperature, counteranions, etc. (Dong *et al.*, 2004; Niu *et al.*, 2009*a*; Sumby & Hardie, 2005; Abu-Youssef *et al.*, 2007). The crystal structures of bis(pyridine-4-carboxylic acid-N)silver(I) nitrate dihydrate (Li *et al.*, 2006), chlorotris(3-methylpyridine-N)silver(I) (Näther & Beck, 2004) and bis[N0-(3-cyanobenzylidene)isonicotinohydrazide]silver(I) trifluoroacetate (Niu *et al.*, 2009*b*) have been reported. We have synthsized a new coordination complex of silver using N'-(2-hydroxybenzylidene)isonicotinohydrazide ligand, (I), and determined its crystal structure which is presented in this article.

The central Ag atom in (I) is coordinated by two nitrogen atoms from two pyridine rings of two different ligands and a water O atom located at the apical site, defining slightly distorted linear coordination geometry (Fig. 1). The cations, anions and solvent water molecules are linked by O—H···O, O—H···N and N—H···O hydrogen bonds into a three-dimensional network (Table 1).

### Experimental

A solution of N'-(2-hydroxybenzylidene)isonicotinohydrazide (0.14 g, 1 mol) in CH_3_OH (10 ml) was added to an aqueous solution of AgNO_3_ (0.1 g, 1 mol) in water (5 ml) with stirring at 333 K. A small amount of precipitate was removed from the resulting solution to grow crystals for crystallographic study. Prism shaped colorless crystals of (I) were obtained by slow evaporation of the solvent from a solution of (I) in CCl_4_ at room temperature over a period of 3 d.

### Refinement

The hydrogen atoms bonded to N and O atoms were located from a difference Fourier map and were allowed to refine freely. The aryl H atoms were placed in calculated position with C—H = 0.95 Å in riding mode, with *U*_iso_(H) = 1.2*U*_eq_ of the carrier C atoms. The residual electron density in the final difference map was located in the close proximity of Ag atom and was essentially meaningless.

### Crystal data

**Table:**

[Ag(C_13_H_11_N_3_O_2_)_2_(H_2_O)]NO_3_	*F*(000) = 1360
*M**_r_* = 670.39	*D*_x_ = 1.673 Mg m^−^^3^
Monoclinic, *P*2_1_/*c*	Mo *K*α radiation, λ = 0.71073 Å
Hall symbol: -P 2ybc	Cell parameters from 530 reflections
*a* = 11.7194 (6) Å	θ = 3–28°
*b* = 12.6459 (6) Å	µ = 0.82 mm^−^^1^
*c* = 18.5719 (9) Å	*T* = 120 K
β = 104.738 (1)°	Prism, colourless
*V* = 2661.8 (2) Å^3^	0.55 × 0.45 × 0.30 mm
*Z* = 4	

### Data collection

**Table:**

Bruker SMART 1000 CCD area-detector diffractometer	6427 independent reflections
Radiation source: normal-focus sealed tube	5518 reflections with *I* > 2σ(*I*)
graphite	*R*_int_ = 0.026
ω scans	θ_max_ = 28.0°, θ_min_ = 1.8°
Absorption correction: multi-scan (*SADABS*; Sheldrick, 1998)	*h* = −15→15
*T*_min_ = 0.686, *T*_max_ = 0.791	*k* = −16→16
26832 measured reflections	*l* = −24→24

### Refinement

**Table:**

Refinement on *F*^2^	Primary atom site location: structure-invariant direct methods
Least-squares matrix: full	Secondary atom site location: difference Fourier map
*R*[*F*^2^ > 2σ(*F*^2^)] = 0.029	Hydrogen site location: difference Fourier map
*wR*(*F*^2^) = 0.077	H atoms treated by a mixture of independent and constrained refinement
*S* = 1.07	*w* = 1/[σ^2^(*F*_o_^2^) + (0.0415*P*)^2^ + 1.2646*P*] where *P* = (*F*_o_^2^ + 2*F*_c_^2^)/3
6427 reflections	(Δ/σ)_max_ = 0.002
403 parameters	Δρ_max_ = 1.04 e Å^−^^3^
0 restraints	Δρ_min_ = −0.57 e Å^−^^3^

### Special details

**Table:**

Geometry. All esds (except the esd in the dihedral angle between two l.s. planes) are estimated using the full covariance matrix. The cell esds are taken into account individually in the estimation of esds in distances, angles and torsion angles; correlations between esds in cell parameters are only used when they are defined by crystal symmetry. An approximate (isotropic) treatment of cell esds is used for estimating esds involving l.s. planes.
Refinement. Refinement of F^2^ against ALL reflections. The weighted R-factor wR and goodness of fit S are based on F^2^, conventional R-factors R are based on F, with F set to zero for negative F^2^. The threshold expression of F^2^ > 2sigma(F^2^) is used only for calculating R-factors(gt) etc. and is not relevant to the choice of reflections for refinement. R-factors based on F^2^ are statistically about twice as large as those based on F, and R- factors based on ALL data will be even larger.

### Fractional atomic coordinates and isotropic or equivalent isotropic displacement parameters (Å^2^)

**Table:**

	*x*	*y*	*z*	*U*_iso_*/*U*_eq_	
Ag1	0.775957 (12)	0.740141 (11)	0.273949 (7)	0.02310 (6)	
O1	0.34784 (12)	0.58945 (10)	−0.05478 (8)	0.0300 (3)	
O2	0.13029 (13)	0.62068 (10)	−0.23755 (8)	0.0270 (3)	
H2O	0.174 (2)	0.642 (2)	−0.1962 (14)	0.039 (7)*	
O3	1.18823 (12)	0.93761 (10)	0.59595 (8)	0.0296 (3)	
O4	1.41552 (13)	0.91337 (11)	0.78672 (8)	0.0281 (3)	
H4O	1.373 (2)	0.8915 (19)	0.7484 (14)	0.036 (7)*	
N1	0.64148 (14)	0.72810 (12)	0.17184 (8)	0.0205 (3)	
N2	0.31651 (14)	0.76550 (12)	−0.06954 (8)	0.0187 (3)	
H2N	0.3355 (19)	0.827 (2)	−0.0542 (12)	0.026 (6)*	
N3	0.23344 (14)	0.74841 (11)	−0.13455 (9)	0.0184 (3)	
N4	0.91745 (13)	0.77455 (12)	0.37111 (8)	0.0196 (3)	
N5	1.23864 (14)	0.76411 (12)	0.61217 (9)	0.0187 (3)	
H5N	1.239 (2)	0.700 (2)	0.5912 (15)	0.045 (7)*	
N6	1.31761 (13)	0.78524 (12)	0.67872 (8)	0.0197 (3)	
C2	0.59564 (16)	0.63071 (15)	0.14847 (10)	0.0246 (4)	
H2A	0.6244	0.5710	0.1787	0.030*	
C3	0.50945 (16)	0.61508 (14)	0.08293 (10)	0.0235 (4)	
H3A	0.4807	0.5460	0.0687	0.028*	
C4	0.46537 (15)	0.70135 (14)	0.03811 (9)	0.0194 (3)	
C5	0.51035 (16)	0.80089 (14)	0.06191 (10)	0.0216 (3)	
H5A	0.4820	0.8618	0.0329	0.026*	
C6	0.59588 (16)	0.81041 (15)	0.12748 (10)	0.0229 (4)	
H6A	0.6248	0.8792	0.1426	0.027*	
C7	0.37251 (15)	0.67941 (14)	−0.03274 (10)	0.0200 (3)	
C8	0.17347 (15)	0.82700 (14)	−0.16895 (9)	0.0194 (3)	
H8A	0.1879	0.8968	−0.1499	0.023*	
C9	0.08375 (15)	0.80705 (14)	−0.23707 (10)	0.0200 (3)	
C10	0.06388 (16)	0.70525 (15)	−0.26912 (10)	0.0210 (3)	
C11	−0.02388 (17)	0.68925 (17)	−0.33365 (10)	0.0273 (4)	
H11A	−0.0355	0.6209	−0.3555	0.033*	
C12	−0.09495 (17)	0.77244 (17)	−0.36661 (11)	0.0280 (4)	
H12A	−0.1553	0.7608	−0.4109	0.034*	
C13	−0.07862 (17)	0.87339 (17)	−0.33525 (10)	0.0281 (4)	
H13A	−0.1282	0.9302	−0.3575	0.034*	
C14	0.01051 (16)	0.88956 (15)	−0.27153 (10)	0.0241 (4)	
H14A	0.0224	0.9584	−0.2506	0.029*	
C16	0.96229 (16)	0.87207 (14)	0.38692 (10)	0.0234 (4)	
H16A	0.9344	0.9272	0.3522	0.028*	
C17	1.04701 (16)	0.89570 (14)	0.45134 (10)	0.0231 (4)	
H19B	1.0772	0.9655	0.4602	0.028*	
C18	1.08769 (15)	0.81621 (14)	0.50305 (9)	0.0194 (3)	
C19	1.04189 (16)	0.71489 (15)	0.48736 (10)	0.0207 (3)	
H19A	1.0678	0.6587	0.5215	0.025*	
C20	0.95837 (16)	0.69751 (14)	0.42146 (10)	0.0211 (3)	
H20A	0.9280	0.6280	0.4109	0.025*	
C21	1.17579 (15)	0.84614 (14)	0.57436 (9)	0.0200 (3)	
C22	1.38215 (15)	0.70897 (14)	0.71241 (10)	0.0200 (3)	
H22A	1.3736	0.6401	0.6913	0.024*	
C23	1.46765 (16)	0.72811 (14)	0.78232 (10)	0.0209 (3)	
C24	1.48193 (16)	0.82865 (15)	0.81647 (10)	0.0224 (4)	
C25	1.56702 (17)	0.84345 (16)	0.88292 (10)	0.0279 (4)	
H25A	1.5760	0.9109	0.9062	0.033*	
C26	1.63897 (18)	0.76048 (17)	0.91552 (11)	0.0303 (4)	
H26A	1.6972	0.7718	0.9608	0.036*	
C27	1.62699 (17)	0.66057 (17)	0.88272 (11)	0.0309 (4)	
H27A	1.6768	0.6040	0.9052	0.037*	
C28	1.54123 (17)	0.64503 (16)	0.81680 (10)	0.0264 (4)	
H28A	1.5320	0.5769	0.7945	0.032*	
N7	0.23978 (14)	0.99376 (11)	−0.00096 (8)	0.0240 (3)	
O5	0.14711 (16)	1.04260 (14)	−0.02193 (9)	0.0520 (5)	
O6	0.31228 (13)	0.98880 (11)	−0.03995 (8)	0.0314 (3)	
O7	0.26376 (13)	0.94629 (10)	0.06137 (7)	0.0284 (3)	
O1W	0.76685 (14)	0.54006 (11)	0.30562 (9)	0.0308 (3)	
H1W	0.759 (2)	0.519 (2)	0.3471 (15)	0.039 (7)*	
H2W	0.801 (3)	0.496 (2)	0.2897 (16)	0.055 (9)*	

### Atomic displacement parameters (Å^2^)

**Table:**

	*U*^11^	*U*^22^	*U*^33^	*U*^12^	*U*^13^	*U*^23^
Ag1	0.01855 (9)	0.03073 (9)	0.01712 (8)	0.00154 (5)	−0.00076 (6)	0.00185 (5)
O1	0.0320 (7)	0.0221 (7)	0.0302 (7)	0.0029 (5)	−0.0025 (6)	−0.0047 (5)
O2	0.0318 (7)	0.0214 (6)	0.0253 (7)	−0.0001 (5)	0.0029 (6)	−0.0062 (5)
O3	0.0332 (7)	0.0218 (7)	0.0290 (7)	0.0022 (5)	−0.0009 (6)	−0.0058 (5)
O4	0.0323 (7)	0.0235 (7)	0.0249 (7)	−0.0031 (5)	0.0005 (6)	−0.0050 (5)
N1	0.0221 (8)	0.0266 (8)	0.0132 (7)	0.0085 (6)	0.0052 (6)	0.0035 (6)
N2	0.0184 (7)	0.0197 (7)	0.0154 (7)	−0.0004 (5)	−0.0008 (6)	−0.0019 (5)
N3	0.0173 (7)	0.0232 (7)	0.0146 (7)	−0.0013 (5)	0.0037 (6)	−0.0008 (5)
N4	0.0150 (7)	0.0264 (8)	0.0164 (7)	0.0022 (6)	0.0021 (6)	0.0014 (6)
N5	0.0191 (7)	0.0195 (7)	0.0159 (7)	−0.0015 (5)	0.0013 (6)	−0.0021 (5)
N6	0.0180 (7)	0.0246 (7)	0.0152 (7)	−0.0021 (6)	0.0020 (6)	−0.0019 (6)
C2	0.0235 (9)	0.0239 (9)	0.0252 (9)	0.0064 (7)	0.0039 (7)	0.0050 (7)
C3	0.0244 (9)	0.0212 (8)	0.0238 (9)	0.0037 (7)	0.0040 (7)	0.0011 (7)
C4	0.0196 (8)	0.0206 (8)	0.0185 (8)	0.0033 (6)	0.0057 (7)	0.0011 (6)
C5	0.0213 (9)	0.0209 (9)	0.0219 (9)	0.0012 (7)	0.0038 (7)	0.0013 (7)
C6	0.0219 (9)	0.0231 (9)	0.0223 (9)	−0.0023 (7)	0.0029 (7)	0.0010 (7)
C7	0.0171 (8)	0.0233 (8)	0.0190 (8)	0.0028 (6)	0.0037 (6)	−0.0002 (6)
C8	0.0198 (8)	0.0198 (8)	0.0178 (8)	−0.0020 (6)	0.0034 (6)	−0.0018 (6)
C9	0.0202 (8)	0.0222 (8)	0.0183 (8)	−0.0035 (7)	0.0057 (7)	−0.0006 (6)
C10	0.0201 (8)	0.0241 (9)	0.0200 (8)	−0.0031 (7)	0.0071 (7)	−0.0011 (7)
C11	0.0253 (9)	0.0357 (11)	0.0205 (9)	−0.0067 (8)	0.0054 (7)	−0.0057 (8)
C12	0.0178 (9)	0.0488 (12)	0.0149 (8)	−0.0055 (8)	−0.0005 (7)	0.0011 (8)
C13	0.0230 (9)	0.0377 (11)	0.0226 (9)	0.0023 (8)	0.0039 (7)	0.0087 (8)
C14	0.0269 (9)	0.0248 (9)	0.0206 (9)	−0.0011 (7)	0.0062 (7)	0.0024 (7)
C16	0.0252 (9)	0.0213 (8)	0.0234 (9)	0.0046 (7)	0.0059 (7)	0.0044 (7)
C17	0.0263 (9)	0.0190 (8)	0.0237 (9)	0.0006 (7)	0.0054 (7)	−0.0001 (7)
C18	0.0192 (8)	0.0207 (8)	0.0189 (8)	0.0020 (6)	0.0060 (7)	−0.0010 (6)
C19	0.0197 (8)	0.0217 (8)	0.0197 (8)	−0.0001 (7)	0.0034 (7)	0.0030 (7)
C20	0.0222 (8)	0.0200 (8)	0.0188 (8)	−0.0012 (7)	0.0014 (7)	0.0012 (6)
C21	0.0196 (8)	0.0213 (8)	0.0192 (8)	0.0003 (6)	0.0053 (7)	−0.0006 (6)
C22	0.0214 (8)	0.0198 (8)	0.0183 (8)	−0.0023 (7)	0.0040 (7)	−0.0009 (6)
C23	0.0191 (8)	0.0260 (9)	0.0174 (8)	−0.0034 (7)	0.0042 (7)	0.0009 (7)
C24	0.0203 (8)	0.0262 (9)	0.0208 (8)	−0.0057 (7)	0.0056 (7)	0.0002 (7)
C25	0.0262 (10)	0.0348 (10)	0.0219 (9)	−0.0109 (8)	0.0046 (7)	−0.0032 (8)
C26	0.0202 (9)	0.0496 (13)	0.0180 (9)	−0.0098 (8)	−0.0006 (7)	0.0044 (8)
C27	0.0241 (10)	0.0411 (11)	0.0261 (10)	0.0006 (8)	0.0036 (8)	0.0104 (8)
C28	0.0261 (9)	0.0283 (9)	0.0242 (9)	0.0001 (7)	0.0052 (7)	0.0046 (7)
N7	0.0332 (8)	0.0160 (7)	0.0185 (7)	0.0010 (6)	−0.0012 (6)	−0.0018 (5)
O5	0.0536 (11)	0.0551 (11)	0.0430 (9)	0.0335 (9)	0.0045 (8)	0.0096 (8)
O6	0.0413 (8)	0.0264 (7)	0.0276 (7)	−0.0055 (6)	0.0108 (6)	−0.0014 (5)
O7	0.0404 (8)	0.0210 (6)	0.0192 (6)	−0.0051 (6)	−0.0007 (6)	0.0023 (5)
O1W	0.0431 (9)	0.0238 (7)	0.0281 (8)	0.0042 (6)	0.0138 (7)	0.0015 (6)

### Geometric parameters (Å, °)

**Table:**

Ag1—N1	2.1406 (16)	C10—C11	1.381 (3)
Ag1—N4	2.1616 (15)	C11—C12	1.384 (3)
Ag1—O1W	2.6059 (14)	C11—H11A	0.9500
O1—C7	1.219 (2)	C12—C13	1.396 (3)
O2—C10	1.364 (2)	C12—H12A	0.9500
O2—H2O	0.85 (3)	C13—C14	1.380 (3)
O3—C21	1.221 (2)	C13—H13A	0.9500
O4—C24	1.356 (2)	C14—H14A	0.9500
O4—H4O	0.81 (3)	C16—C17	1.379 (3)
N1—C6	1.350 (2)	C16—H16A	0.9500
N1—C2	1.369 (2)	C17—C18	1.388 (2)
N2—N3	1.362 (2)	C17—H19B	0.9500
N2—C7	1.362 (2)	C18—C19	1.391 (3)
N2—H2N	0.83 (2)	C18—C21	1.506 (2)
N3—C8	1.289 (2)	C19—C20	1.377 (2)
N4—C16	1.343 (2)	C19—H19A	0.9500
N4—C20	1.351 (2)	C20—H20A	0.9500
N5—C21	1.360 (2)	C22—C23	1.444 (3)
N5—N6	1.369 (2)	C22—H22A	0.9500
N5—H5N	0.90 (3)	C23—C28	1.406 (3)
N6—C22	1.286 (2)	C23—C24	1.412 (3)
C2—C3	1.384 (3)	C24—C25	1.388 (3)
C2—H2A	0.9500	C25—C26	1.385 (3)
C3—C4	1.390 (2)	C25—H25A	0.9500
C3—H3A	0.9500	C26—C27	1.394 (3)
C4—C5	1.393 (3)	C26—H26A	0.9500
C4—C7	1.505 (2)	C27—C28	1.386 (3)
C5—C6	1.372 (2)	C27—H27A	0.9500
C5—H5A	0.9500	C28—H28A	0.9500
C6—H6A	0.9500	N7—O5	1.224 (2)
C8—C9	1.447 (2)	N7—O6	1.250 (2)
C8—H8A	0.9500	N7—O7	1.2705 (19)
C9—C14	1.398 (2)	O1W—H1W	0.84 (3)
C9—C10	1.412 (3)	O1W—H2W	0.79 (3)
			
N1—Ag1—N4	171.63 (6)	C13—C12—H12A	119.8
N1—Ag1—O1W	93.88 (5)	C14—C13—C12	119.12 (18)
N4—Ag1—O1W	94.23 (5)	C14—C13—H13A	120.4
C10—O2—H2O	106.6 (17)	C12—C13—H13A	120.4
C24—O4—H4O	104.5 (18)	C13—C14—C9	121.63 (18)
C6—N1—C2	115.94 (16)	C13—C14—H14A	119.2
C6—N1—Ag1	125.00 (13)	C9—C14—H14A	119.2
C2—N1—Ag1	119.07 (11)	N4—C16—C17	122.94 (16)
N3—N2—C7	117.57 (15)	N4—C16—H16A	118.5
N3—N2—H2N	121.2 (15)	C17—C16—H16A	118.5
C7—N2—H2N	121.1 (15)	C16—C17—C18	119.21 (17)
C8—N3—N2	119.60 (14)	C16—C17—H19B	120.4
C16—N4—C20	117.42 (16)	C18—C17—H19B	120.4
C16—N4—Ag1	122.79 (12)	C17—C18—C19	118.41 (16)
C20—N4—Ag1	119.63 (12)	C17—C18—C21	117.67 (16)
C21—N5—N6	118.07 (15)	C19—C18—C21	123.88 (16)
C21—N5—H5N	121.6 (17)	C20—C19—C18	118.85 (17)
N6—N5—H5N	119.4 (17)	C20—C19—H19A	120.6
C22—N6—N5	118.16 (15)	C18—C19—H19A	120.6
N1—C2—C3	123.16 (16)	N4—C20—C19	123.17 (17)
N1—C2—H2A	118.4	N4—C20—H20A	118.4
C3—C2—H2A	118.4	C19—C20—H20A	118.4
C2—C3—C4	119.45 (17)	O3—C21—N5	123.33 (16)
C2—C3—H3A	120.3	O3—C21—C18	121.66 (16)
C4—C3—H3A	120.3	N5—C21—C18	115.00 (15)
C3—C4—C5	117.81 (17)	N6—C22—C23	119.96 (16)
C3—C4—C7	117.06 (16)	N6—C22—H22A	120.0
C5—C4—C7	125.13 (16)	C23—C22—H22A	120.0
C6—C5—C4	119.55 (17)	C28—C23—C24	118.66 (17)
C6—C5—H5A	120.2	C28—C23—C22	119.27 (17)
C4—C5—H5A	120.2	C24—C23—C22	122.05 (17)
N1—C6—C5	124.09 (17)	O4—C24—C25	117.62 (17)
N1—C6—H6A	118.0	O4—C24—C23	122.56 (16)
C5—C6—H6A	118.0	C25—C24—C23	119.82 (18)
O1—C7—N2	122.33 (16)	C26—C25—C24	120.42 (19)
O1—C7—C4	121.53 (16)	C26—C25—H25A	119.8
N2—C7—C4	116.13 (15)	C24—C25—H25A	119.8
N3—C8—C9	118.84 (16)	C25—C26—C27	120.87 (19)
N3—C8—H8A	120.6	C25—C26—H26A	119.6
C9—C8—H8A	120.6	C27—C26—H26A	119.6
C14—C9—C10	118.10 (16)	C28—C27—C26	118.96 (19)
C14—C9—C8	119.67 (16)	C28—C27—H27A	120.5
C10—C9—C8	122.18 (16)	C26—C27—H27A	120.5
O2—C10—C11	118.38 (17)	C27—C28—C23	121.28 (19)
O2—C10—C9	121.24 (16)	C27—C28—H28A	119.4
C11—C10—C9	120.38 (18)	C23—C28—H28A	119.4
C10—C11—C12	120.25 (19)	O5—N7—O6	121.33 (16)
C10—C11—H11A	119.9	O5—N7—O7	120.08 (17)
C12—C11—H11A	119.9	O6—N7—O7	118.59 (15)
C11—C12—C13	120.50 (18)	H1W—O1W—H2W	108 (3)
C11—C12—H12A	119.8		
			
O1W—Ag1—N1—C6	175.36 (14)	C12—C13—C14—C9	−0.9 (3)
O1W—Ag1—N1—C2	−4.07 (14)	C10—C9—C14—C13	−0.3 (3)
O1W—Ag1—N4—C16	−178.91 (14)	C8—C9—C14—C13	−177.92 (17)
O1W—Ag1—N4—C20	−3.61 (14)	C20—N4—C16—C17	0.1 (3)
C7—N2—N3—C8	175.98 (16)	Ag1—N4—C16—C17	175.52 (14)
C21—N5—N6—C22	−175.93 (16)	N4—C16—C17—C18	−0.7 (3)
C6—N1—C2—C3	1.2 (3)	C16—C17—C18—C19	0.6 (3)
Ag1—N1—C2—C3	−179.31 (14)	C16—C17—C18—C21	−177.08 (16)
N1—C2—C3—C4	−0.5 (3)	C17—C18—C19—C20	0.0 (3)
C2—C3—C4—C5	−0.3 (3)	C21—C18—C19—C20	177.56 (17)
C2—C3—C4—C7	179.53 (16)	C16—N4—C20—C19	0.6 (3)
C3—C4—C5—C6	0.4 (3)	Ag1—N4—C20—C19	−174.99 (14)
C7—C4—C5—C6	−179.42 (17)	C18—C19—C20—N4	−0.6 (3)
C2—N1—C6—C5	−1.1 (3)	N6—N5—C21—O3	1.7 (3)
Ag1—N1—C6—C5	179.44 (14)	N6—N5—C21—C18	−177.92 (14)
C4—C5—C6—N1	0.3 (3)	C17—C18—C21—O3	19.3 (3)
N3—N2—C7—O1	−2.5 (3)	C19—C18—C21—O3	−158.27 (18)
N3—N2—C7—C4	178.87 (15)	C17—C18—C21—N5	−161.04 (16)
C3—C4—C7—O1	−9.2 (3)	C19—C18—C21—N5	21.4 (2)
C5—C4—C7—O1	170.63 (17)	N5—N6—C22—C23	179.24 (16)
C3—C4—C7—N2	169.47 (16)	N6—C22—C23—C28	−177.15 (17)
C5—C4—C7—N2	−10.7 (3)	N6—C22—C23—C24	1.2 (3)
N2—N3—C8—C9	−178.63 (15)	C28—C23—C24—O4	179.40 (17)
N3—C8—C9—C14	174.16 (16)	C22—C23—C24—O4	1.0 (3)
N3—C8—C9—C10	−3.3 (3)	C28—C23—C24—C25	−0.3 (3)
C14—C9—C10—O2	−178.56 (16)	C22—C23—C24—C25	−178.70 (17)
C8—C9—C10—O2	−1.0 (3)	O4—C24—C25—C26	−178.97 (17)
C14—C9—C10—C11	1.5 (3)	C23—C24—C25—C26	0.8 (3)
C8—C9—C10—C11	179.04 (17)	C24—C25—C26—C27	−0.4 (3)
O2—C10—C11—C12	178.61 (16)	C25—C26—C27—C28	−0.3 (3)
C9—C10—C11—C12	−1.5 (3)	C26—C27—C28—C23	0.8 (3)
C10—C11—C12—C13	0.2 (3)	C24—C23—C28—C27	−0.4 (3)
C11—C12—C13—C14	1.0 (3)	C22—C23—C28—C27	177.98 (17)

### Hydrogen-bond geometry (Å, °)

**Table:**

*D*—H···*A*	*D*—H	H···*A*	*D*···*A*	*D*—H···*A*
O1W—H1W···O7^i^	0.84 (3)	2.01 (3)	2.844 (2)	171 (3)
O1W—H2W···O2^ii^	0.79 (3)	2.04 (3)	2.821 (2)	172 (3)
N2—H2N···O6	0.84 (3)	2.09 (3)	2.880 (2)	157 (2)
N5—H5N···O7^iii^	0.90 (3)	1.97 (3)	2.863 (2)	169 (2)
O2—H2O···N3	0.85 (3)	1.79 (3)	2.560 (2)	150 (2)
O4—H4O···N6	0.81 (2)	1.86 (2)	2.607 (2)	153 (2)

Symmetry codes: (i) −*x*+1, *y*−1/2, −*z*+1/2; (ii) −*x*+1, −*y*+1, −*z*; (iii) *x*+1, −*y*+3/2, *z*+1/2.

## References

[bb1] Abu-Youssef, M. A. M., Dey, R., Gohar, Y., Massoud, A. A., Ohrstrom, L. & Langer, V. (2007). *Inorg. Chem.* **46**, 5893–5903. 10.1021/ic0621594 17602550

[bb2] Bruker (2007). *SMART* and *SAINT-Plus* Bruker AXS, Madison, Wisconsin, USA.

[bb3] Dong, Y.-B., Zhao, X., Huang, R.-Q., Smith, M. D. & zur Loye, H.-C. (2004). *Inorg. Chem.* **43**, 5603–5612. 10.1021/ic049787a 15332812

[bb4] Li, B., Gao, P., Ye, L., Yang, G.-D. & Wu, L.-X. (2006). *Acta Cryst.* E**62**, m3238–m3239.

[bb5] Näther, C. & Beck, A. (2004). *Acta Cryst.* E**60**, m1678–m1680.

[bb6] Niu, C.-Y., Wu, B.-L., Zheng, X.-F., Wan, X.-S., Zhang, H.-Y., Niu, Y.-Y. & Meng, L.-Y. (2009*a*). *CrystEngComm*, **11**, 1373–1382.

[bb7] Niu, C.-Y., Zhang, H.-Y. & Wan, X.-S. (2009*b*). *Acta Cryst.* E**65**, m1285. 10.1107/S1600536809039579 PMC2971296 21578055

[bb8] Sheldrick, G. M. (1998). *SADABS.* University of Göttingen, Germany.

[bb9] Sheldrick, G. M. (2008). *Acta Cryst.* A**64**, 112–122. 10.1107/S0108767307043930 18156677

[bb10] Sumby, C. J. & Hardie, M. J. (2005). *Angew. Chem. Int. Ed.* **44**, 6395–6399. 10.1002/anie.200501358 16145700

